# The association between postoperative myocardial injury of unexplained aetiology after noncardiac surgery and sex and cancer on 1-yr survival: a retrospective, single-centre, observational cohort study

**DOI:** 10.1016/j.bjao.2025.100485

**Published:** 2025-09-29

**Authors:** Eva P.C. van Schaik, Lisette M. Vernooij, Younes Haddou, Remco B. Grobben, Wilton A. van Klei, Judith A.R. van Waes

**Affiliations:** 1 Department of Anaesthesiology and Intensive Care Medicine, University Medical Center Utrecht, University Utrecht, Utrecht, The Netherlands; 2 Department of Anaesthesiology, Intensive Care and Pain Medicine, St Antonius Hospital, Nieuwegein, The Netherlands; 3 Department of Anaesthesiology and Pain Medicine, Antoni van Leeuwenhoek, Amsterdam, The Netherlands; 4 Department of Cardiology, University Medical Center Utrecht, University Utrecht, Utrecht, The Netherlands; 5 Department of Cardiology, Amphia Hospital, Breda, The Netherlands; 6 Department of Anaesthesia and Pain Management, Toronto General Hospital, University Health Network Toronto, ON, Canada; 7 Department of Anaesthesiology and Pain Medicine, Temerty Faculty of Medicine, University of Toronto, Toronto, ON, Canada; 8 Toronto General Hospital Research Institute, Toronto, ON, Canada

**Keywords:** cardiac optimisation prognosis, noncardiac surgery, postoperative myocardial injury, sex differences, troponins

## Abstract

**Background:**

The aetiology of postoperative myocardial injury (PMI) is often unexplained, and the effect of sex and cancer surgery on prognosis in patients with PMI is unknown. We aimed to estimate the proportion of patients developing PMI of unknown aetiology and compare their prognosis with those with explained PMI, and to investigate the interaction with sex and cancer surgery.

**Methods:**

This retrospective, single-centre, observational cohort study included patients aged ≥60 yr undergoing noncardiac surgery. Patients with PMI (defined as an elevated troponin concentration <72h after surgery) were categorised into five groups, based on the most likely aetiology of PMI: acute myocardial infarction (MI); extra-cardiac disease (acute or chronic renal failure, sepsis, pulmonary embolism, acute intracranial pathology, or all of the mentioned); known cardiac disease with regular follow-up; perioperative haemodynamic/respiratory events; and PMI of unexplained aetiology. The association between PMI group and 1-yr mortality, and between sex and cancer surgery, was estimated.

**Results:**

Of 3885 patients, 823 (21%) had a diagnosis of PMI, of whom 32 (4%) had MI, 201 (24%) had extra-cardiac disease, 174 (21%) had known cardiac disease, 269 (33%) had haemodynamic/respiratory events, and 147 (18%) had PMI of unexplained aetiology. Like other patients with PMI, those with PMI of unexplained aetiology had an increased risk of 1-yr mortality (risk ratio 1.5, 95% confidence interval 1.1–2.1). PMI caused by MI or known cardiac disease occurred more often in men. Women more often had PMI owing to perioperative haemodynamic/respiratory factors. There was no interaction found between PMI and sex or cancer surgery on mortality risk.

**Conclusions:**

In approximately half of the patients with a diagnosis of PMI, PMI aetiology was either related to perioperative haemodynamic/respiratory events or the aetiology was unexplained. These patients had an increased associated mortality risk. Women more often had PMI of likely haemodynamic/respiratory causes. Mortality risk in those with PMI was similar between sexes and between those undergoing cancer surgery *vs* non-cancer surgery.

Postoperative myocardial injury (PMI) frequently occurs after noncardiac surgery with a reported incidence of 9–19%.^1^, ^2^, ^3^ The risk of mortality and morbidity after noncardiac surgery is increased in patients with PMI compared with patients without.^2^^,^^4^^,^^5^ The aetiology of PMI is commonly categorised into subtypes.^6^, ^7^, ^8^ First, PMI may be caused by myocardial ischaemia, either owing to acute coronary syndrome (type 1 ischaemia) or resulting from perioperative events leading to an imbalance in oxygen demand and supply (type 2 ischaemia), and may lead to myocardial infarction (MI).^6^^,^^9^ Second, PMI can also arise from non-ischaemic cardiac causes, such as acute heart failure, tachyarrhythmias, or cardiomyopathy.^9^ Furthermore, PMI can occur in extra-cardiac conditions causing cardiac stress or affecting troponin release, such as sepsis, intracranial pathology, pulmonary embolism (PE) or renal failure. It has been argued that troponin may also be released into the blood stream as a result of cardiomyocyte stress or as consequence of cardiotoxicity caused by certain medications.^7^^,^^10^^,^^11^ The cause of PMI in an individual patient may be multifactorial and is often difficult to diagnose in daily practice.

The majority of patients with PMI are assumed to have type 2 ischaemia owing to perioperative haemodynamic events in the presence of underlying stable coronary artery disease or other cardiac disease. Therapeutical options, however, are generally limited in these patients. First, there often is a relative contraindication for (dual) antiplatelet therapy in the immediate postoperative period. Second, a majority of patients already receives antiplatelet, statin and beta-blocker therapy.^7^^,^^12^, ^13^, ^14^ Consequently, patients who could potentially benefit from cardiac evaluation are those with PMI, but without known cardiovascular disease who do not yet receive primary preventive treatment for adverse cardiovascular events.

Importantly, although sex is a known modifier in cardiovascular disease and symptom presentation, there is limited research on the impact of sex on PMI.^15^ In addition, both PMI and cancer are important predictors of survival, but the interaction between these has not yet been investigated.^16^

The aim of this study was to estimate the proportion of patients developing PMI of unexplained aetiology (i.e. those who may benefit from postoperative cardiac evaluation) and to compare their 1-yr mortality with that of patients in whom a likely explanation for PMI was available. Furthermore, we aimed to evaluate the impact of sex and cancer surgery on the association of PMI of unexplained aetiology and 1-yr mortality.

## Methods

### Study design and patients

This retrospective observational cohort study included consecutive patients aged ≥60 yr who underwent intermediate- to high-risk noncardiac surgery between 1 January 2017 and 31 December 2018 at the University Medical Center Utrecht, The Netherlands. Part of this cohort was used in a previous publication.^17^ Patients who died within 24 h after surgery or who were transferred to another hospital within 24 h were excluded. If a patient underwent surgery more than once within the study period, only the first surgery was included. According to the Institutional Review Board of the University Medical Center Utrecht, the study was not subject to the Medical Research Involving Human Subjects Act (protocol number 21-351/C). This study was conducted in adherence to the STROBE statement for observational research.^18^

### Routine troponin monitoring protocol

According to local protocol, troponin I (TnI) was measured on the first 3 postoperative days. Because of a change in laboratory facilities, two different troponin assays were used during the study period with the following cut-off values: before May 2018, the cut-off value for elevated TnI was ≥60 ng L^–1^ (AccuTnI assay; Beckman Coulter, Brea, CA, USA), and from May 2018 onwards, the cut-off value for elevated high-sensitive TnI was ≥18 ng L^–1^ (Unicel DxI 800; Beckman Coulter). PMI was defined as an elevated TnI above the clinical cut-off value. Before surgery, TnI was not routinely measured.

In case of TnI being elevated, an anaesthesiologist was primarily notified and thereafter a local protocol was activated.^17^ This protocol included additional follow-up TnI sampling every 6 h until values start decreasing, a baseline ECG, laboratory evaluation of haemoglobin values and kidney function, and specific strategies aiming to optimise myocardial oxygen supply and demand (e.g. treating anaemia, hypoxaemia, hypotension, and tachycardia). The protocol further advised to consult a cardiologist in case of symptoms or signs suggestive of acute MI or heart failure, to consult a pulmonologist in case of clinical suspicion of PE, and to consider follow-up at the outpatient cardiac clinic and prescription of antiplatelet therapy, a statin, beta-blockade, or all of the mentioned in patients with high suspicion of myocardial ischaemia.

### Classification of aetiology of Postoperative Myocardial Injury

Patients with PMI were categorised into five groups, based on the most likely cause of PMI and potential benefit from postoperative additional cardiac evaluation.

Group 1: PMI in presence of acute MI, defined as clinically diagnosed by a cardiologist and according to the fourth universal definition^9^; both ST and non-ST segment elevation MI were eligible for this group. As these patients received acute cardiac evaluation, treatment, and long-term follow-up, a separate referral for outpatient cardiac evaluation after discharge seemed redundant.
Group 2: PMI in presence of active extra-cardiac disease, including acute or chronic renal failure (chronic renal failure with preoperative creatinine >177 mM and acute renal failure as defined by the Acute Kidney Injury Network^19^), sepsis as clinically diagnosed by the treating physician, PE and acute intracranial pathology, both radiologically confirmed. Because PMI was secondary to extra-cardiac disease and these patients received treatment and follow-up for this condition, they were considered not likely to benefit from outpatient cardiac evaluation after discharge.
Group 3: PMI in presence of known cardiac disease with recent (<1 yr) follow-up by a cardiologist, including coronary artery disease, valvular disease, arrythmias, and heart failure. The predominant mechanism is deemed type 2 ischaemia, with or without the occurrence of haemodynamic events. We considered that a recent visit to a cardiologist implied that these patients were optimally treated before surgery, and were therefore considered not likely to benefit from additional cardiac evaluation after discharge.
Group 4: PMI after perioperative haemodynamic or respiratory events lasting at least 10 consecutive min, occurring within the time frame from start of surgery up to 3 days after surgery and before the occurrence of PMI. Perioperative events included hypotension with a MAP <65 mm Hg, tachycardia defined as a heart rate >100 beats min^–1^, and anaemia defined as haemoglobin <6.0 mM or hypoxaemia with oxygen saturation <88%.^20^ These patients were either not known with cardiac disease, or had known cardiac disease but without recent (<1 yr) follow-up. We considered that unknown or under-treated cardiac disease may have been present in these patients and that this may have provoked PMI in presence of haemodynamic/respiratory factors (i.e. owing to type 2 ischaemia). Therefore, these patients potentially could benefit from additional (outpatient) cardiac evaluation and treatment.
Group 5: PMI of unexplained aetiology, defined as absence of any of the above four conditions, that is, PMI of unknown cause. We considered that unknown underlying cardiac disease may have caused PMI in these patients, and therefore these patients potentially could benefit from additional cardiac evaluation and treatment after discharge.

**Fig 1:**
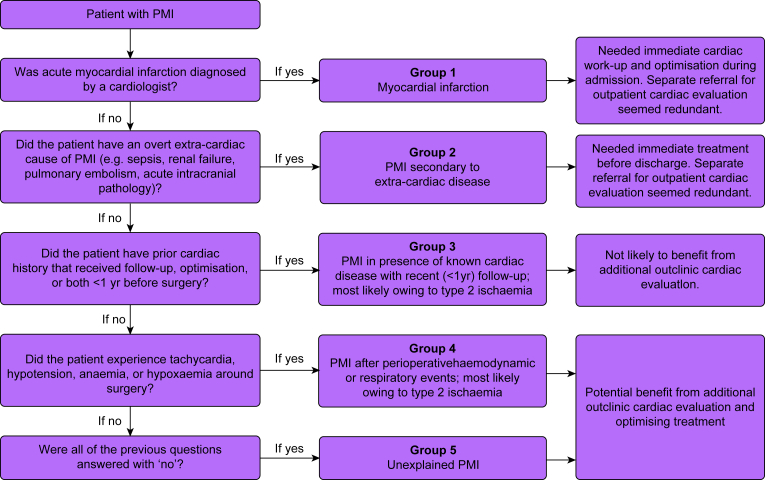
Flowchart showing classification steps of the postoperative myocardial injury (PMI) subgroups. Patients were classified according to the most likely explanation of PMI.

Patients who could be classified into multiple groups were classified to the group that came first in order as pointed out in the flowchart (Fig. 1). For example, a patient with moderate aortic stenosis (Group 3) who had hypotension during surgery (Group 4), was classified to the group ‘known cardiac disease’ (Group 3). This workflow was chosen to first discriminate patients with conditions requiring immediate treatment (e.g. MI, PE, intracranial pathology, or sepsis), and subsequently to identify patients who likely would not benefit from cardiac optimisation (e.g. those with PMI caused by renal disease or by known cardiac disease with recent follow-up). Patients remaining were allocated to Group 4 (perioperative haemodynamic/respiratory events) or Group 5 (unexplained PMI), who potentially could benefit from cardiac evaluation. Further, patients with prior cardiac history who had cardiac follow-up >1 yr ago were not classified as Group 3 and may have been classified as Group 5 despite their cardiac history. This was chosen because we considered that these patients could benefit from cardiac optimisation; for example, a patient who developed PMI in absence of a clear explanation and who was discharged from cardiology follow-up after undergoing a percutaneous coronary intervention 10 yr ago was considered to likely benefit from renewed cardiac optimisation. Uncertainties in group classification were discussed among three authors (ES, YH, JW).

### Outcome

The primary outcome was 1-yr mortality after intermediate- to high-risk noncardiac surgery in patients diagnosed with PMI of unexplained aetiology. Mortality data were collected by consulting the Dutch Personal Records Database.^21^

### Data collection

Data were collected from electronic medical records, including patient and surgery characteristics, comorbidities, vital parameters, laboratory values, and perioperative complications. Missing data were retrieved through medical record screening.

### Statistical analysis

Continuous variables were presented as means (standard deviation [sd]) if normally distributed, or medians (interquartile range [IQR]) if non-normally distributed. All other variables were presented as counts (percentages). Survival was compared between all PMI subgroups and patients without PMI using Kaplan–Meier analysis with log-rank testing and χ^2^ testing to calculate risk ratios (RRs) from contingency tables. To further explore differences in survival between the PMI subgroups, log-rank testing was performed to compare the survival rates of all PMI subgroups with each other, correcting for multiple testing using the Benjamini–Hochberg method.^22^

Next, the proportion of patients classified in the five PMI subgroups was compared between men and women, and between patients undergoing cancer surgery and non-cancer surgery using χ^2^ testing. χ^2^ testing additionally assessed if mortality between men and women and between surgery for cancer and other surgery differed in patients with unexplained PMI. As the rare disease assumption would not hold (incidence of 1-yr mortality was >10%),^23^ a Poisson regression model with robust standard errors was used to investigate the association between the PMI subgroups and 1-yr mortality. Effect estimates were presented as RR with accompanying 95% confidence intervals (CIs). Multivariable analyses were conducted by including *a priori* selected covariates to the model (i.e. age [continuous], sex [dichotomous], and cancer surgery [dichotomous]). Subsequently, to examine if the association differed by sex, an interaction term between sex and PMI subgroup was incorporated in the model. The analytical approach was repeated for assessing the interaction between cancer surgery and PMI subgroup. A *P*-value of <0.05 was considered statistically significant. Analyses were performed in SPSS version 29 (IBM, Chicago, IL, USA) and R software version 2023.12.1.402 (R foundation for Statistical Computing, Vienna, Austria, 2016).

## Results

A total of 3885 patients were included, of whom 823 (21%) had PMI (Table 1). Of all patients with PMI, 32 (4%) had an MI (Group 1), 201 (24%) had PMI that was most likely owing to extra-cardiac disease (Group 2), 174 (21%) had PMI in presence of known cardiac disease (Group 3), 269 patients (33%) had PMI associated with perioperative haemodynamic/respiratory events (Group 4), and 147 patients (18%) had PMI of unexplained aetiology (Group 5). Overall, mean age was 71 yr (range 60-100) and 2181 (66%) were men. Prior cardiac history with last follow-up >1 yr ago was present in 39 (27%) of patients with unexplained PMI (Group 5) and in 86 (32%) patients with PMI owing to haemodynamic events (Group 4). In patients with PMI owing to haemodynamic/respiratory events (Group 4), patients frequently underwent general surgery (35%), including both cancer and non-cancer surgery. Baseline characteristics stratified for sex are presented in Supplementary Tables 1 and 2.

**Table 1:** Baseline characteristics of patients with postoperative myocardial injury (PMI), stratified in subgroups, and those without PMI. IQR, inter-quartile range; sd, standard deviation.

	All	PMI subgroup					No PMI
		Group 1	Group 2	Group 3	Group 4	Group 5	
		Myocardial infarction	PMI owing to extra-cardiac disease	PMI owing to known cardiac disease	PMI owing to haemodynamic events	Unexplained PMI	
	*N*=3885	*N*=32	*N*=201	*N*=174	*N*=269	*N*=147	*N*=3062
	*n* (%)	*n* (%)	*n* (%)	*n* (%)	*n* (%)	*n* (%)	*n* (%)
**Age (yr), mean (range)**	71 (60–100)	75 (61–86)	74 (60–100)	75 (60–96)	74 (60–97)	74 (61–93)	70 (60–94)
**Female**	1704 (44)	6 (19)	83 (41)	53 (31)	121 (45)	51 (35)	1390 (45)
**Revised Cardiac Risk Index**							
0	1528 (39)	5 (16)	18 (9)	18 (10)	82 (29)	60 (39)	1350 (44)
1	1482 (38)	7 (22)	107 (53)	42 (24)	124 (44)	53 (34)	1154 (38)
2	605 (16)	10 (31)	55 (27)	54 (31)	57 (20)	31 (20)	405 (13)
≥3	270 (7)	10 (31)	24 (12)	60 (35)	17 (6)	10 (7)	153 (5)
**Cardiac history**	1164 (30)	20 (62)	82 (41)	174 (100)	86 (32)	39 (27)	763 (25)
Ischaemic disease	596 (15)	15 (47)	40 (20)	106 (61)	42 (16)	20 (14)	373 (12)
Heart failure	293 (8)	1 (3)	13 (7)	43 (25)	9 (3)	3 (2)	224 (7)
Valvular disease	429 (11)	8 (25)	21 (10)	99 (57)	18 (7)	11 (8)	272 (9)
Arrhythmia	512 (13)	5 (16)	43 (21)	64 (37)	42 (16)	17 (12)	341 (11)
Other cardiac disease	100 (3)	1 (3)	14 (7)	38 (22)	9 (3)	7 (5)	31 (1)
**Preoperative creatinine >177 μmol L^–1^**	159 (4)	3 (9)	26 (13)	34 (20)	23 (9)	5 (3)	68 (2)
**Hypertension**	1946 (50)	25 (78)	122 (61)	122 (70)	160 (60)	85 (58)	1432 (47)
**Vascular disease**	706 (18)	20 (63)	60 (30)	72 (41)	55 (20)	32 (22)	467 (15)
**Pulmonary disease**	916 (24)	7 (22)	37 (18)	57 (33)	67 (25)	25 (17)	723 (24)
**Diabetes Mellitus**	606 (16)	10 (31)	38 (19)	51 (29)	50 (19)	28 (19)	429 (14)
**Cerebrovascular disease**	610 (16)	12 (38)	98 (49)	44 (25)	34 (13)	28 (19)	394 (13)
**Cancer surgery**	1701 (44)	5 (16)	46 (23)	51 (29)	111 (41)	70 (48)	1418 (46)
**Surgical specialty**							
General	927 (24)	5 (16)	57 (28)	48 (28)	93 (35)	35 (24)	689 (23)
Neurosurgical	638 (16)	5 (16)	86 (43)	11 (6)	21 (8)	30 (20)	485 (16)
Head and neck	772 (20)	2 (6)	5 (3)	15 (9)	27 (10)	26 (18)	697 (23)
Gynaecological	301 (8)	0	2 (1)	3 (2)	11 (4)	7 (5)	278 (9)
Orthopaedic	384 (10)	2 (6)	9 (5)	22 (13)	54 (20)	17 (12)	280 (9)
Vascular	507 (13)	16 (50)	30 (15)	61 (35)	49 (18)	22 (15)	329 (11)
Urological	356 (9)	2 (6)	12 (6)	14 (8)	14 (5)	10 (7)	304 (10)
**Duration of surgery (min), median (IQR)**	155 (95–238)	216 (104–330)	144 (78–259)	161 (98–241)	212 (131–320)	182 (120–306)	151 (93–227)

### Survival within Postoperative Myocardial Injury subgroups

**Fig 2:**
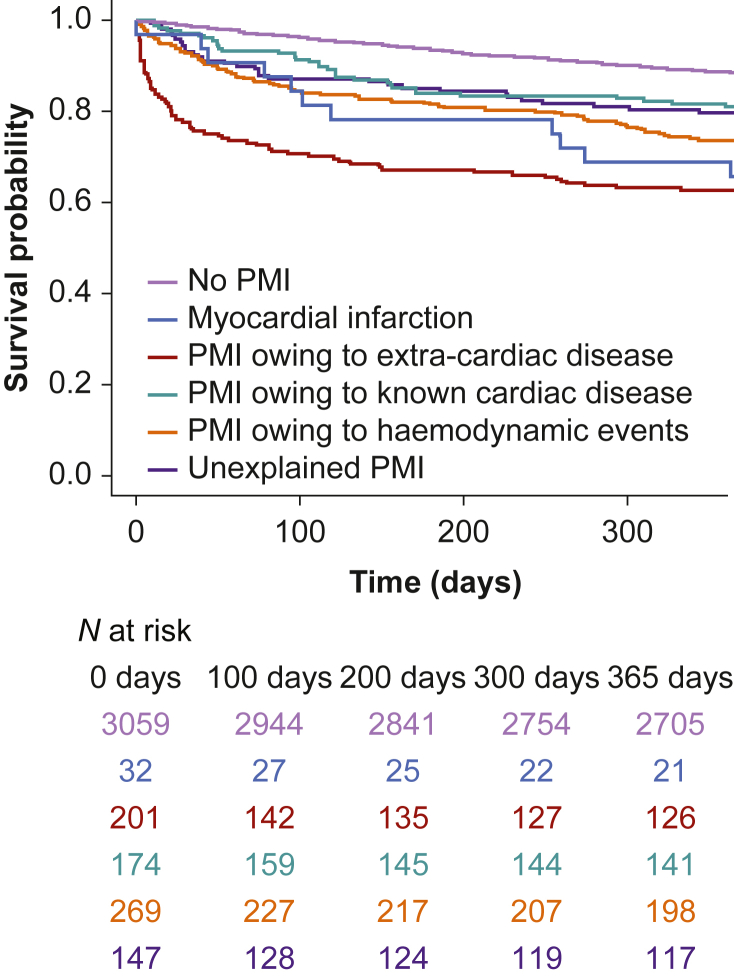
Kaplan–Meier plot of patients by postoperative myocardial injury (PMI) subgroup and patients without PMI. Mortality was measured as 1-yr mortality.

Of the 823 patients with PMI, 220 (26%) died within 1 yr after surgery compared with 354 (12%) of the 3062 patients without PMI (RR 2.3, 95% CI 2.0–2.7; Table 2). The highest mortality rates were observed in patients with PMI resulting from extra-cardiac conditions (Group 2; *n*=75, 37%) and MI (Group 1; *n*=11, 34%). Survival in all PMI groups differed significantly from patients without PMI (Fig. 2, Supplementary Table 3). Among patients with PMI, survival of patients with PMI of unexplained aetiology (Group 5) did not significantly differ from patients with PMI resulting from MI (Group 1), known cardiac disease (Group 3), or haemodynamic/respiratory events (Group 4). Patients with unexplained PMI (Group 5) had a comparable risk of mortality (RR 1.5, 95% CI 1.1–2.2) as patients with PMI owing to known cardiac disease (Group 3; RR 1.6, 95% CI 1.2–2.3) and patients with PMI owing to haemodynamic/respiratory events (Group 4; RR 2.1, 95% CI 1.7–2.7; Table 3).

**Table 2:** One-year mortality in the postoperative myocardial injury (PMI) subgroups compared with patients without PMI stratified by sex and indication for surgery (cancer *vs* non-cancer).

	No PMI	Group 1	Group 2	Group 3	Group 4	Group 5
		Myocardial infarction	PMI owing to extra-cardiac disease	PMI owing to known cardiac disease	PMI owing to haemodynamic events	Unexplained PMI
	(*N*=3062)	(*N*=32)	(*N*=201)	(*N*=174)	(*N*=269)	(*N*=147)
	*n* (%)	*n* (%)	*n* (%)	*n* (%)	*n* (%)	*n* (%)
**1-yr mortality stratified by:**						
All patients	354 (12)	11 (34)	75 (37)	33 (19)	71 (26)	30 (20)
Women (*N*=1704)	142 (5)	2 (6)	28 (14)	13 (7)	26 (10)	10 (7)
Men (*N*=2181)	212 (7)	9 (28)	47 (23)	20 (12)	45 (17)	20 (14)
Cancer surgery (*N*=1701)	263 (9)	2 (6)	17 (8)	12 (7)	32 (12)	22 (15)
Non-cancer surgery (*N*=2184)	91 (3)	9 (28)	58 (29)	21 (12)	39 (14)	8 (5)

**Table 3:** Poisson regression analysis for the association between postoperative myocardial injury (PMI) subgroup and 1-yr mortality adjusted for age, sex, and cancer surgery. CI, confidence interval; RR, risk ratio.

Variable		RR (95% CI)	*P*-value
**Age**		1.03 (1.02–1.04)	<0.001
**Female sex**		0.85 (0.73–0.99)	0.033
**Cancer surgery**		2.29 (1.97–2.66)	<0.001

**PMI group**			
	No PMI	ref	ref

Group 1	Myocardial infarction	3.43 (2.11–5.58)	<0.001
Group 2	Owing to extra-cardiac disease	3.59 (2.89–4.46)	<0.001
Group 3	Owing to known cardiac disease	1.62 (1.17–2.25)	0.004
Group 4	Owing to haemodynamic events	2.14 (1.71–2.68)	<0.001
Group 5	Unexplained	1.54 (1.11–2.13)	0.009

### Sex-related differences among Postoperative Myocardial Injury subgroups

**Fig 3:**
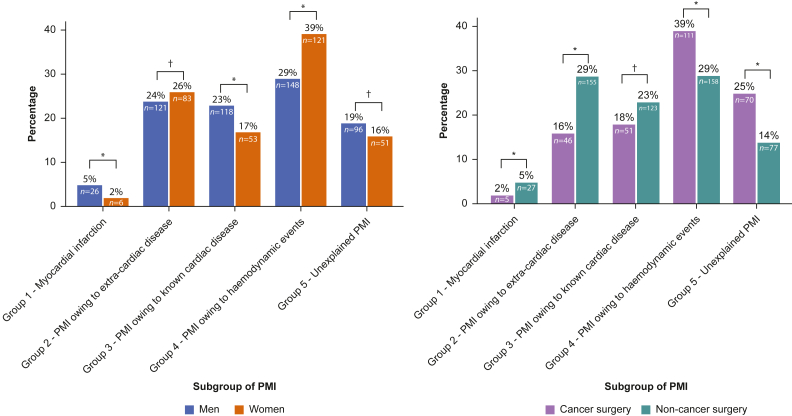
Bar chart showing the distribution of patients by postoperative myocardial injury (PMI) subgroup according to sex and cancer surgery. *Significant dfference between groups (P<0.05). ^†^No significant dfference between groups (P>0.05).

PMI occurred in 314 (18%) of 1704 women *vs* 509 (23%) of 2181 men; RR 0.8 (95% CI 0.7–0.9). The proportion of patients with unexplained PMI (Group 5) did not differ among men (19%, *N*=96) and women (16%, *N*=51); RR 1.2 (95% CI 0.9–1.6). PMI owing to MI (Group 1) occurred more often in men (5%, *N*=26) than in women (2%, *N*=6); RR 2.7 (95% CI 1.1–6.4), and men more often had PMI owing to known cardiac disease (Group 3; 24%, *N*=121) compared with women (17%, *N*=53); RR 1.4 (95% CI 1.1–1.9) (Fig. 3). Women more often had PMI resulting from perioperative haemodynamic/respiratory factors (Group 4; *N*=121, 39%) than men (*N*=148, 29%); RR 1.3 (95% CI 1.1–1.6). Kaplan–Meier plots for mortality stratified by sex are presented in Supplementary Figure 1. Mortality rates of men with unexplained PMI (Group 5; *N*=20, 21%) and women with unexplained PMI (*N*=10, 20%) were comparable (RR 1.1, 95% CI 0.9–1.4).There was no interaction between sex and PMI subgroup on 1-yr mortality (Supplementary Table 4).

### Subgroup analysis for cancer and non-cancer surgery patients

PMI occurred in 283 (17%) of the 1701 patients undergoing cancer surgery compared with 540 (25%) of the 2184 patients undergoing non-cancer surgery; RR 0.7 (95% CI 0.6–0.8). Patients with PMI undergoing cancer surgery more often had unexplained PMI (Group 5; 25%, *N*=70) and PMI resulting from haemodynamic/respiratory events (Group 4; 39%, *N*=111) compared with patients with PMI undergoing non-cancer surgery (14%, *N*=77 and 29%, *N*=158, respectively), with corresponding RRs of 1.7 (95% CI 1.3–2.3) and 1.3 (95% CI 1.1–1.6), respectively (Fig. 3). Mortality rates were higher in patients with unexplained PMI (Group 5) after cancer surgery (*N*=22, 31%) than in patients with unexplained PMI after non-cancer surgery (*N*=8, 10%); RR 3.0 (95% CI 1.4–6.4) (Table 2, Supplementary Fig. 2). There was interaction between cancer surgery and PMI in Groups 2, 3, and 4 (Supplementary Table 5).

## Discussion

In a cohort of patients undergoing noncardiac surgery, we aimed to estimate the proportion of patients developing PMI of unexplained aetiology who could benefit from postoperative cardiac evaluation. We also compared their prognosis with that of patients in whom a likely explanation for PMI was available, and we evaluated the impact of sex and cancer on the association between unexplained PMI and mortality. Approximately half of the patients with PMI (i.e. those with PMI owing to haemodynamic/respiratory events and those with PMI of unexplained aetiology) could potentially benefit from additional (outpatient) cardiac evaluation after surgery. All-cause mortality was highest in patients with extra-cardiac causes (including acute or chronic renal failure, sepsis, PE, acute intracranial pathology, or all of the mentioned). The mortality risk in patients with PMI of unexplained aetiology and PMI owing to perioperative haemodynamic/respiratory events was comparable with those with PMI owing to known cardiac disease with recent follow-up. Compared with men, women more often had PMI owing to perioperative haemodynamic/respiratory events in absence of diagnosed cardiac disease. The risk of PMI on mortality was similar for men and women.

In patients with PMI, asymptomatic obstructive coronary artery disease has been shown to be prevalent (50%), indicating that type 2 ischaemia is the predominant aetiologic mechanism of PMI.^24^^,^^25^ Similar data was observed by the BASEL-PMI study group who reported that in 73% of patients with PMI this was likely owing to type 2 ischaemia. Of note, 40% of these patients were known with coronary artery disease.^13^ In patients with PMI of unexplained aetiology, extra-cardiac pathology such as asymptomatic PE may also be present.^24^^,^^25^ Moreover, PMI can also occur as a physiological response to stress or inflammation.^10^ This latter mechanism is supported by the finding that neither obstructive coronary artery disease nor PE is present in a significant number of patients with PMI.^24^^,^^25^

It is important to note that the overall mortality risk in patients with PMI mainly is not driven by patients who could benefit from additional cardiac evaluation, but rather by patients with severe extra-cardiac causes of PMI (Group 2). This potentially hampers the effect on prognosis that may be achieved by cardiac optimisation strategies. This is in line with the limited efficacy of preventive strategies in prior trials in noncardiac surgical populations including the POISE and MANAGE trials.^26^, ^27^, ^28^ Furthermore, it underlines the relevance of selection of surgical populations at high risk for PMI to justify routine troponin monitoring, especially with current staffing shortages and increasing healthcare costs.^29^^,^^30^ Further investigation should therefore determine whether underlying, undiagnosed cardiac disease is present in the subgroup of PMI patients who are deemed to have benefit from cardiac evaluation and optimisation.

Research on sex-specific differences in PMI is still limited. In our study, the proportion of patients with PMI of unexplained aetiology (Group 5) did not differ between men and women. Although women had an overall lower risk of mortality, this risk was independent of PMI subgroup. A previous study showed that women had a lower risk of overall mortality after PMI (hazard ratio 0.8, 95% CI 0.7–0.8).^31^ From a biological perspective, men more often have ischaemic heart disease, whereas women more often are diagnosed with heart failure or microvascular heart disease.^32^ As microvascular disease may lead to a poor compensatory response to haemodynamic events, this might initiate type 2 ischaemia in women. In our study, PMI owing to haemodynamic events either in absence of diagnosed cardiac disease, or with cardiac disease but without recent follow-up (Group 4) occurred more in women, whereas the risk of PMI on mortality was similar for both sexes. This suggests that cardiac disease in women either occurs less frequently, or may be underdiagnosed, as cardiovascular disease in women is overlooked because of different and delayed symptom presentation.^32^^,^^33^

Compared with patients undergoing non-cancer surgery, patients undergoing cancer surgery more often had PMI of unexplained aetiology (Group 5), and less frequently had PMI owing to known cardiac disease (Group 3). This may be explained by the fact that patients undergoing non-cancer surgery, such as vascular surgery and orthopaedic surgery, generally have more comorbidities such as coronary artery disease and diabetes mellitus than patients undergoing cancer surgery.^34^^,^^35^ PMI in patients undergoing cancer surgery might be caused by noncardiac, undiagnosed pathology (such as asymptomatic PE or chemotherapy-induced cardiotoxicity).^36^^,^^37^ Furthermore, our findings suggest that patients undergoing cancer surgeries experienced a lower risk of death from PMI than those undergoing non-cancer surgeries. The higher mortality rates that were observed in patients undergoing cancer surgery are therefore likely more attributable to their cancer diagnosis than PMI. Oncological prognosis should therefore be taken into account when cardiac evaluation is considered in patients with PMI after cancer surgery.

Our study has several important limitations, mainly because of the retrospective nature. Firstly, the exact aetiologic mechanism behind PMI was often not known, and perioperative haemodynamic/respiratory events may have been missed as postoperative vital parameters were not continuously measured up to the third postoperative day. Secondly, despite following a predefined workflow to classify patients according to the most likely explanation for PMI by experienced clinical researchers, misclassification may have occurred. We would like to emphasise that this classification was not created to point out the exact aetiology of PMI, but rather to distinguish the patients with PMI who may benefit from additional postoperative (outpatient) cardiac evaluation, from those who will likely not. Because the aetiology of PMI is multifactorial and there was no established PMI classification for our aim, we created these five groups. We acknowledge that this classification is not suitable for classification of the aetiology of PMI. Nevertheless, we believe that this pragmatic classification may shed light on the usefulness of troponin screening and may aid future research investigating the efficacy of treatment or follow-up of PMI. Third, as causes of death were unknown, we can only hypothesise that mortality in cancer surgery patients was more likely owing to their cancer than to PMI. Fourthly, because troponin was not measured before surgery and we could not distinguish acute from chronic elevations, all patients with elevated postoperative troponin concentrations were classified as having PMI, and patients with chronic troponin elevations may have been misclassified. Fifthly, as we did not use sex-specific cut-off points for troponin, which are lower for women, we may have underestimated the proportion of women with PMI. Sixthly, this study was conducted in a tertiary referral hospital where major surgeries, including a high percentage of cancer surgery and emergency procedures such as acute neurosurgical interventions are performed; therefore, the results of our study may not be generalisable to other centres. This may also have led to relatively larger groups of patients with PMI owing to known cardiac disease or (acute) extra-cardiac causes when compared with non-academic hospitals. Finally, the PMI subgroups had relatively small sample sizes, which may have resulted in underpowered analyses.

## Conclusion

In approximately half of the patients with a diagnosis of postoperative myocardial injury (defined as an elevated TnI above the clinical cut-off value >72 h after surgery), PMI was associated with perioperative haemodynamic/respiratory events or was of unexplained aetiology. As these two patient groups potentially could benefit from cardiac work-up, the added value of postoperative cardiac evaluation to identify and optimise underlying cardiac disease in those patients should be topic of further research. Women more often had PMI owing to perioperative haemodynamic/respiratory events in absence of diagnosed cardiac disease compared with men. However, the risk of PMI on mortality was similar for men and women, potentially suggesting the presence of undiagnosed cardiac disease in women. We report no association between PMI and cancer surgery.

## Authors’ contributions

Study conception/design: EVS LMV, JvW, RBG, WAvK

Data acquisition: EVS, YH, LMV, JvW

Data analysis: EVS, LMV, JvW, WAvK

Data interpretation: EVS, LMV, JvW, RBG, WAvK

Drafting of paper: EVS, LMV, YH, JvW, WAvK, RBG

Critical revision of paper for important intellectual content: all authors

Final approval of paper: all authors

All authors agree to be accountable for all aspects of the work in ensuring that questions related to the accuracy or integrity of any part of the work are appropriately investigated and resolved.

## Declaration of interests

The authors declare that they have no conflicts of interest.
