# Supplementary material for: The association between postoperative myocardial injury of unexplained aetiology after noncardiac surgery and sex and cancer on 1-yr survival: a retrospective, single-centre, observational cohort study
Source: BJA Open. 2025 Sep 29;16:100485. doi: 10.1016/j.bjao.2025.100485 (PMC12513324; doi:10.1016/j.bjao.2025.100485)
Supplement: Multimedia component 1 [file mmc1.docx]

|  | **All** | **PMI subgroup** | | | | | **No PMI** |
| --- | --- | --- | --- | --- | --- | --- | --- |
|  |  | Group 1 | Group 2 | Group 3 | Group 4 | Group 5 |  |
|  |  | Myocardial infarction | PMI owing to extra-cardiac disease | PMI owing to known cardiac disease | PMI owing to haemodynamic events | Unexplained PMI |  |
|  | N= 2181 | N=26 | n=118 | n=121 | n=148 | n=96 | n=1672 |
|  | **n(%)** | **n(%)** | **n(%)** | **n(%)** | **n(%)** | **n(%)** | **n(%)** |
| **Mean age (years, range)** | 71 (60-100) | 75 (61-86) | 75 (61-100) | 74 (61-91) | 74 (60-97) | 74 (61-91) | 70 (60-94) |
| **Revised Cardiac Risk Index** |  |  |  |  |  |  |  |
| *0* | 794 (36) | 3 (12) | 5 (4) | 10 (8) | 34(23) | 40 (42) | 702 (42) |
| *1* | 792 (36) | 5 (19) | 59 (50) | 26 (22) | 63 (43) | 32 (33) | 607 (36) |
| *2* | 394 (18) | 9 (35) | 35 (30) | 37 (31) | 42 (28) | 17 (18) | 254 (15) |
| *3 or more* | 201 (9) | 9 (35) | 19 (16) | 48 (39) | 9 (6) | 7 (7) | 109 (7) |
| **Cardiac history** | 791 (36) | 18 (69) | 56 (47) | 121 (100) | 59 (40) | 31 (32) | 506 (30) |
| *Ischaemic disease* | 461 (21) | 14 (54) | 31 (26) | 88 (73) | 32 (22) | 15 (16) | 281 (17) |
| *Heart failure* | 187 (9) | 1 (4) | 8 (7) | 30 (25) | 7 (5) | 2 (2) | 139 (8) |
| *Valvular disease* | 247 (11) | 7 (27) | 10 (9) | 57 (47) | 9 (6) | 5 (5) | 159 (10) |
| *Arrhythmia* | 358 (16) | 4 (15) | 32 (27) | 44 (36) | 28 (19) | 14 (15) | 236 (14) |
| *Other cardiac disease* | 61 (3) | 1 (4) | 8 (7) | 19 (16) | 7 (5) | 7 (7) | 19 (1) |
| **Creatinine  >177µmol L^-1^** | 116 (5) | 2 (8) | 21 (18) | 25 (21) | 18 (12) | 4 (4) | 46 (3) |
| **Hypertension** | 1106 (51) | 19 (73) | 76 (64) | 81 (67) | 93 (63) | 52 (54) | 785 (47) |
| **Vascular disease** | 481 (22) | 19 (69) | 33 (28) | 60 (50) | 36 (24) | 25 (26) | 308 (18) |
| **Pulmonary disease** | 529 (24) | 6 (23) | 24 (20) | 43 (36) | 35 (24) | 16 (17) | 405 (24) |
| **Diabetes Mellitus** | 359 (17) | 8 (31) | 22 (19) | 34 (28) | 28 (19) | 14 (15) | 253 (15) |
| **Cerebrovascular disease** | 380 (17) | 10 (39) | 56 (48) | 30 (25) | 24 (16) | 20 (21) | 240 (14) |
| **Cancer surgery** | 960 (44) | 3 (12) | 28 (24) | 34 (28) | 71 (48) | 43 (45) | 781 (47) |
| **Surgical specialty** |  |  |  |  |  |  |  |
| *General* | 511 (23) | 4 (15) | 33 (28) | 31 (26) | 56 (38) | 17 (18) | 370 (22) |
| *Neurosurgical* | 356 (16) | 4 (15) | 47 (40) | 8 (7) | 12 (8) | 23 (24) | 262 (16) |
| *Head and Neck* | 496 (23) | 1 (4) | 3 (3) | 10 (8) | 19 (13) | 19 (20) | 444 (27) |
| *Gynaecological* | 0 | 0 | 0 | 0 | 0 | 0 | 0 |
| *Orthopaedic* | 180 (8) | 1 (4) | 7 (6) | 14 (12) | 22 (15) | 11 (12) | 125 (8) |
| *Vascular* | 353 (16) | 14 (54) | 20 (17) | 47 (39) | 31 (21) | 17 (18) | 224 (13) |
| *Urological* | 285 (13) | 2 (8) | 8 (7) | 11 (9) | 8 (5) | 9 (9) | 247 (15) |
| **Median duration of surgery in minutes (IQR)** | 158 (93-246) | 204 (100-305) | 132 (79-258) | 164 (100-279) | 208 (140-347) | 187 (130-298) | 152 (90-230) |

**Supplement Table 1. Baseline demographics in men with PMI, stratified in subgroups, and men without PMI**

Legend: Abbreviations: IQR: interquartile range, µmol/L: micromoles per litre, PMI: postoperative myocardial injury, SD: standard deviation.

**Supplement Table 2. Baseline demographics in women with PMI, stratified in subgroups, and women without PMI**

Legend: Abbreviations: IQR: interquartile range, µmol/L: micromoles per litre, PMI: postoperative myocardial injury, SD: standard deviation.

|  | **All** | **PMI subgroup** | | | | | **No PMI** |
| --- | --- | --- | --- | --- | --- | --- | --- |
|  |  | Group 1 | Group 2 | Group 3 | Group 4 | Group 5 |  |
|  |  | Myocardial infarction | PMI owing to extra-cardiac disease | PMI owing to known cardiac disease | PMI owing to haemodynamic events | Unexplained PMI |  |
|  | N=1704 | N=6 | n=83 | n= 53 | n=121 | n=51 | n=1390 |
|  | **n(%)** | **n(%)** | **n(%)** | **n(%)** | **n(%)** | **n(%)** | **n(%)** |
| **Mean age (years, range)** | 71 (60-96) | 74 (67-81) | 72 (60-91) | 77 (60-96) | 73 (61-94) | 75 (61-93) | 70 (60-93) |
| **Revised Cardiac Risk Index** |  |  |  |  |  |  |  |
| *0* | 734 (43) | 2 (33) | 12 (15) | 8 (15) | 45 (37) | 19 (37) | 648 (47) |
| *1* | 690 (41) | 2 (33) | 46 (55) | 16 (30) | 60 (50) | 19 (37) | 547 (39) |
| *2* | 211 (12) | 1 (17) | 20 (24) | 17 (32) | 10 (8) | 12 (24) | 151 (11) |
| *3 or more* | 69 (4) | 1 (17) | 5 (6) | 12 (13) | 6 (5) | 1 (2) | 44 (3) |
| **Cardiac history** | 374 (22) | 2 (33) | 27 (33) | 53 (100) | 27 (22) | 8 (16) | 257 (19) |
| *Ischaemic disease* | 135 (8) | 1 (17) | 9 (11) | 18 (34) | 10 (8) | 5 (10) | 92 (7) |
| *Heart failure* | 106 (6) | 0 | 5 (6) | 13 (25) | 2 (2) | 1 (2) | 85 (6) |
| *Valvular disease* | 182 (11) | 1 (17) | 11 (13) | 42 (79) | 9 (7) | 6 (12) | 113 (81) |
| *Arrhythmia* | 154 (9) | 1 (17) | 11 (13) | 20 (38) | 14 (12) | 3 (6) | 105 (8) |
| *Other cardiac disease* | 39 (2) | 0 | 6 (7) | 19 (36) | 2 (2) | 0 | 12 (1) |
| **Creatinine >177µmol L^-1^** | 43 (3) | 1 (17) | 5 (6) | 9 (17) | 5 (4) | 1 (2) | 22 (2) |
| **Hypertension** | 840 (49) | 6 (100) | 46 (55) | 41 (77) | 67 (55) | 33 (65) | 647 (46) |
| **Vascular disease** | 226 (13) | 2 (33) | 27 (33) | 12 (23) | 19 (16) | 7 (14) | 159 (11) |
| **Pulmonary disease** | 387 (23) | 1 (17) | 13 (16) | 14 (26) | 32 (26) | 9 (18) | 318 (23) |
| **Diabetes Mellitus** | 247 (15) | 2 (33) | 16 (19) | 17 (32) | 22 (18) | 14 (28) | 176 (13) |
| **Cerebrovascular disease** | 230 (14) | 2 (33) | 42 (51) | 14 (26) | 10 (8) | 8 (16) | 154 (11) |
| **Cancer surgery** | 741 (44) | 2 (33) | 18 (22) | 17 (32) | 40 (33) | 27 (53) | 637 (46) |
| **Surgical specialty** |  |  |  |  |  |  |  |
| *General* | 416 (24) | 1 (17) | 24 (29) | 17 (32) | 37 (31) | 18 (35) | 319 (23) |
| *Neurosurgical* | 282 (17) | 1 (17) | 39 (47) | 3 (6) | 9 (7) | 7 (14) | 223 (16) |
| *Head and Neck* | 276 (16) | 1 (17) | 2 (2) | 5 (9) | 8 (7) | 7 (14) | 253 (18) |
| *Gynaecological* | 301 (18) | 0 | 2 (2) | 3 (6) | 11 (9) | 7 (14) | 278 (20) |
| *Orthopaedic* | 204 (12) | 1 (17) | 2 (2) | 8 (15) | 32 (26) | 6 (12) | 155 (11) |
| *Vascular* | 154 (9) | 2 (33) | 10 (12) | 14 (16) | 18 (15) | 5 (10) | 105 (8) |
| *Urological* | 71 (4) | 0 | 4 (5) | 3 (6) | 6 (5) | 1 (2) | 57 (4) |
| **Median duration of surgery in minutes (IQR)** | 153 (95-232) | 300 (136-382) | 149 (75-264) | 158 (91-188) | 214 (129-298) | 179 (103-308) | 148 (95-225) |

|  |  |  | Group 1 | Group 2 | Group 3 | Group 4 | Group 5 |
| --- | --- | --- | --- | --- | --- | --- | --- |
|  |  | **No PMI** | **Myocardial infarction** | **PMI owing to extra-cardiac disease** | **PMI owing to known cardiac disease** | **PMI owing to haemodynamic events** | **Unexplained PMI** |
| Group 1 | **Myocardial infarction** | p <0.001 | - | - | - | - | - |
| Group 2 | **PMI owing to extra-cardiac disease** | p <0.001 | P = 0.561 | - | - | - | - |
| Group 3 | **PMI owing to known cardiac disease** | p =0.004 | P = 0.080 | P <0.001 | - | - | - |
| Group 4 | **PMI owing to haemodynamic events** | p <0.001 | P = 0.439 | P =0.005 | P = 0.100 | - | - |
| Group 5 | **Unexplained PMI** | p =0.001 | P = 0.152 | P <0.001 | P = 0.706 | P = 0.230 | - |

**Supplement Table 3. Benjamin Hochberg posthoc analysis of survival of patients with PMI in different subgroups and patients without PMI.**

**Supplement table 4.** **Poisson regression analysis of the association between PMI subgroup and one-year mortality, adjusted for age and cancer surgery, and including an interaction term for PMI subgroup and sex.**

|  |  | **RR (95%CI)** | **P value** |
| --- | --- | --- | --- |
| **Age** |  | 1.03 (1.02-1.04) | <0.001 |
| **Female sex** |  | 0.81 (0.66-0.98) | 0.03 |
| **Cancer surgery** |  | 2.28 (1.96-2.65) | <0.001 |
| **PMI group** |  |  |  |
|  | No PMI | ref | ref |
| Group 1 | Myocardial infarction | 3.38 (2.01-5.70) | 0.001 |
| Group 2 | Owing to extra-cardiac disease | 3.42 (2.61-4.51) | <0.001 |
| Group 3 | Owing to known cardiac disease | 1.37 (0.90-2.08) | 0.141 |
| Group 4 | Owing to haemodynamic events | 2.11 (1.58-2.80) | <0.001 |
| Group 5 | Unexplained | 1.50 (1.01-2.25) | 0.046 |
| **Interaction between**  **PMI group and sex** | |  |  |
|  |  |  |  |
|  | No PMI * female sex | ref | ref |
| Group 1 | Myocardial infarction  * female sex | 1.02 (0.25-4.09) | 0.981 |
| Group 2 | Owing to extra-cardiac disease * female sex | 1.13 (0.73-1.75) | 0.584 |
| Group 3 | Owing to known cardiac disease * female sex | 1.63 (0.84-3.16) | 0.148 |
| Group 4 | Owing to haemodynamic events * female sex | 1.04 (0.65-1.65) | 0.880 |
| Group 5 | Unexplained * female sex | 1.06 (0.54-2.08) | 0.861 |

Legend: RR = risk ratio, 95% CI = 95% confidence interval.

**Supplement table 5. Poisson regression analysis of the association between PMI subgroup and one-year mortality, adjusted for age and sex, and including an interaction term for PMI subgroup and cancer surgery.**

| **Variable** |  |  | **RR (95%CI)** | **P value** |
| --- | --- | --- | --- | --- |
| **Age** |  |  | 1.03 (1.02-1.04) | <0.001 |
| **Female sex** |  |  | 0.84 (0.72-0.97) | 0.022 |
| **Cancer surgery** |  |  | 3.32 (2.65-4.17) | <0.001 |
| **PMI group** |  |  |  |  |
|  | No PMI |  | ref | ref |
| Group 1 | Myocardial infarction |  | 4.96 (2.80-8.76) | <0.001 |
| Group 2 | Owing to extra-cardiac disease |  | 5.93 (4.41-7.98) | <0.001 |
| Group 3 | Owing to known cardiac disease |  | 2.67 (1.70-4.18) | <0.001 |
| Group 4 | Owing to haemodynamic events |  | 4.05 (2.90-5.67) | <0.001 |
| Group 5 | Unexplained |  | 1.63 (0.82-3.23) | 0.163 |
| **Interaction between**  **PMI group and cancer surgery** | |  |  |  |
|  |  |  |  |  |
|  | No PMI * cancer surgery |  | ref | ref |
| Group 1 | Myocardial infarction * cancer surgery |  | 0.43 (0.13-1.38) | 0.157 |
| Group 2 | Owing to extra-cardiac disease * cancer surgery |  | 0.32 (0.20-0.52) | <0.001 |
| Group 3 | Owing to known cardiac disease * cancer surgery |  | 0.40 (0.21-0.78) | 0.007 |
| Group 4 | Owing to haemodynamic events * cancer surgery |  | 0.34 (0.22-0.54) | <0.001 |
| Group 5 | Unexplained * cancer surgery |  | 0.93 (0.43-2.01) | 0.852 |

Legend: RR = risk ratio, 95% CI = 95% confidence interval.

**Supplement Figure 1. Kaplan Meier plots stratified by sex and PMI.**


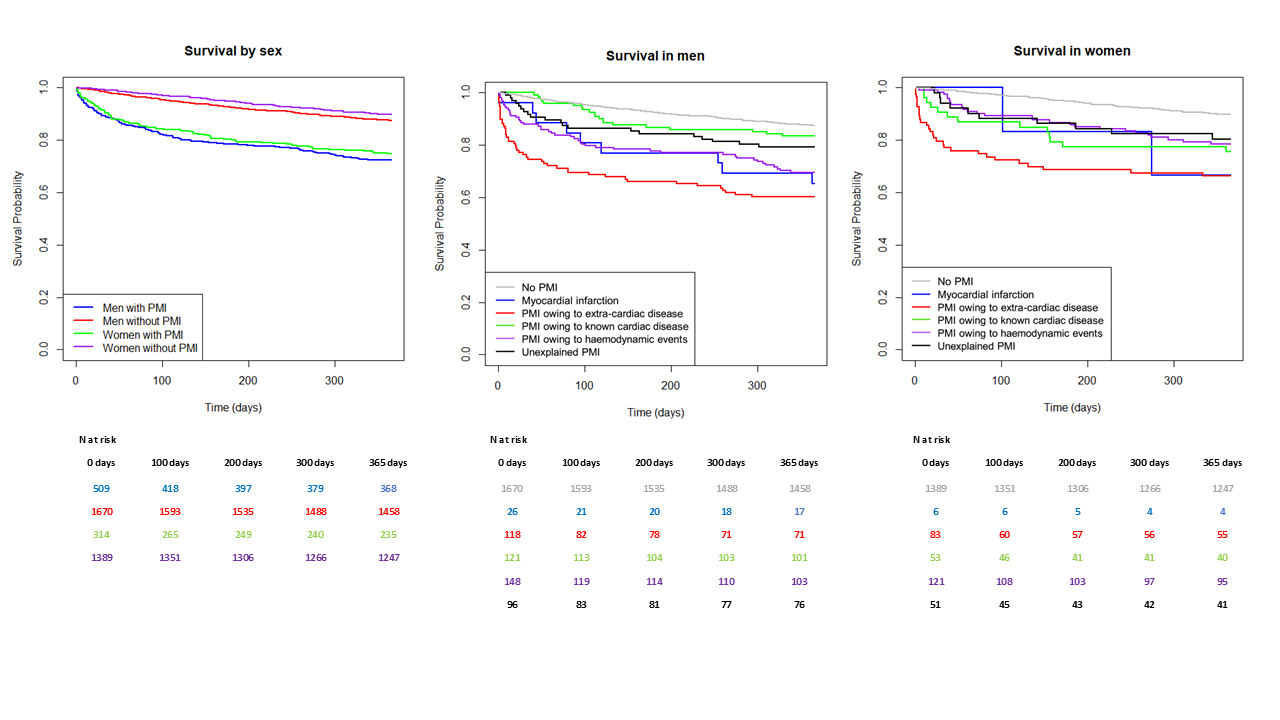


Figure legend:

Mortality was measured as one-year mortality.

Left: Kaplan Meier plot in both men and women with and without PMI.
Middle: Kaplan meier plot of men with PMI, stratified by subgroup, and without PMI.
Right = Kaplan meier plot of women with PMI, stratified by subgroup, and without PMI.

**Supplement Figure 2**. **Kaplan Meier plots stratified by cancer surgery and PMI.**


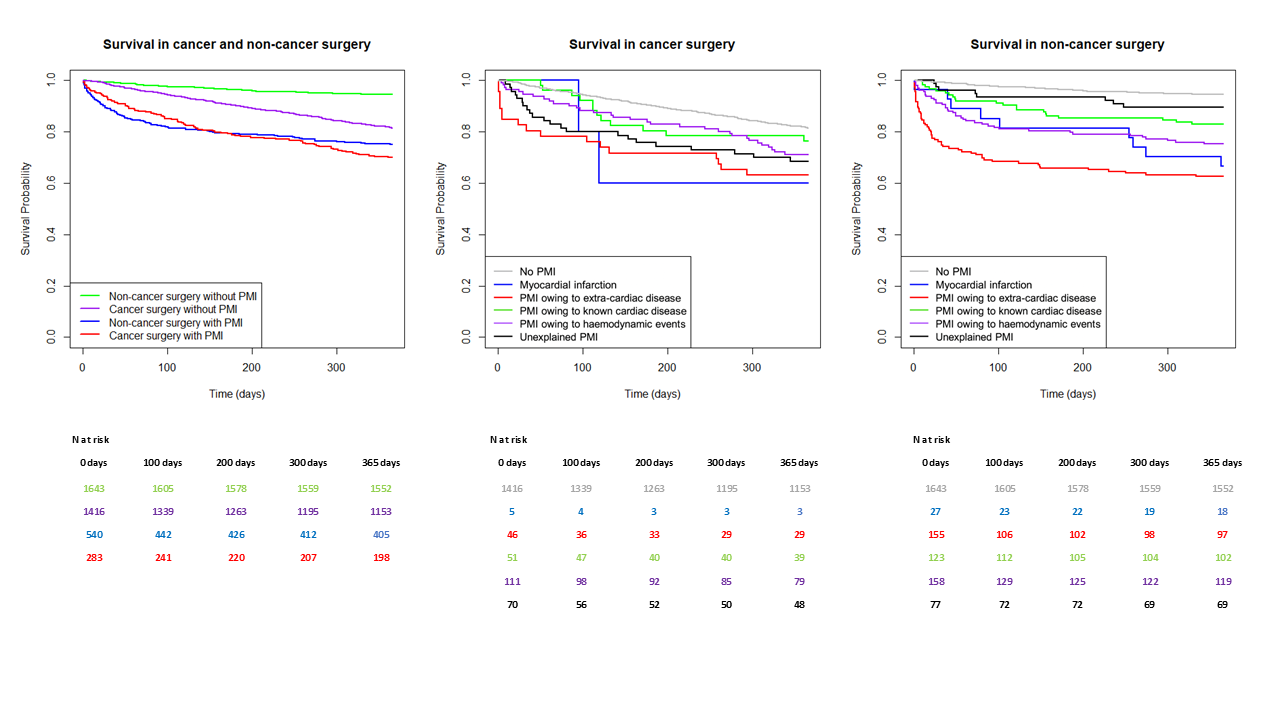


Figure legend:

Mortality was measured as one-year mortality.

Left: Kaplan Meier plot in patients with cancer and non-cancer surgery with and without PMI.
Middle: Kaplan meier plot of patients with cancer surgery with PMI, stratified by subgroup, and without PMI.
Right = Kaplan meier plot of patients with non-cancer surgery with PMI, stratified by subgroup, and without PMI

Page intentionally left blank
